# Using test-time augmentation to investigate explainable AI: inconsistencies between method, model and human intuition

**DOI:** 10.1186/s13321-024-00824-1

**Published:** 2024-04-04

**Authors:** Peter B. R. Hartog, Fabian Krüger, Samuel Genheden, Igor V. Tetko

**Affiliations:** 1 Molecular AI, Discovery Sciences, R &D, AstraZeneca, 431 83 Mölndal, Sweden; 2 Institute of Structural Biology, Helmholtz Munich, Munich, 85764 Germany

**Keywords:** ML, XAI, Robustness, Test-time augmentation, Interpretation, Representation learning, Explainability

## Abstract

**Abstract:**

Stakeholders of machine learning models desire explainable artificial intelligence (XAI) to produce human-understandable and consistent interpretations. In computational toxicity, augmentation of text-based molecular representations has been used successfully for transfer learning on downstream tasks. Augmentations of molecular representations can also be used at inference to compare differences between multiple representations of the same ground-truth. In this study, we investigate the robustness of eight XAI methods using test-time augmentation for a molecular-representation model in the field of computational toxicity prediction. We report significant differences between explanations for different representations of the same ground-truth, and show that randomized models have similar variance. We hypothesize that text-based molecular representations in this and past research reflect tokenization more than learned parameters. Furthermore, we see a greater variance between in-domain predictions than out-of-domain predictions, indicating XAI measures something other than learned parameters. Finally, we investigate the relative importance given to expert-derived structural alerts and find similar importance given irregardless of applicability domain, randomization and varying training procedures. We therefore caution future research to validate their methods using a similar comparison to human intuition without further investigation.

**Scientific contribution:**

In this research we critically investigate XAI through test-time augmentation, contrasting previous assumptions about using expert validation and showing inconsistencies within models for identical representations. SMILES augmentation has been used to increase model accuracy, but was here adapted from the field of image test-time augmentation to be used as an independent indication of the consistency within SMILES-based molecular representation models.

**Graphical Abstract:**

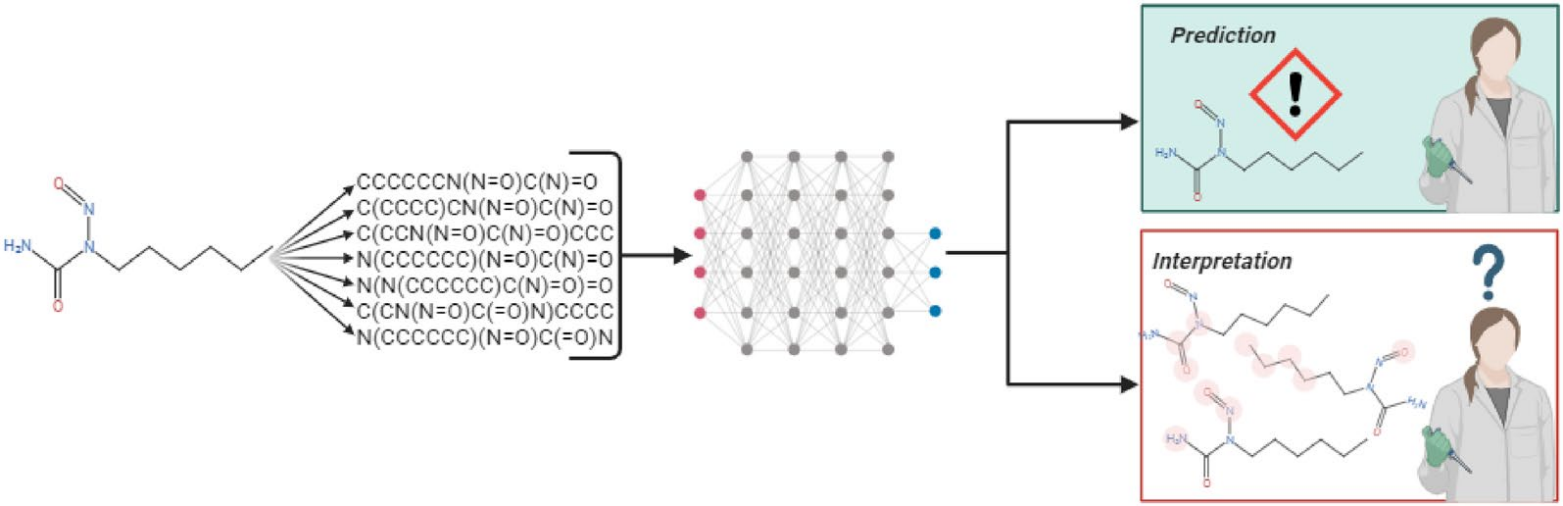

## Introduction

Explainable artificial intelligence (XAI) is used to elucidate how predictions from machine learning (ML) models are generated [1]. XAI is required to identify security and bias risks, enhance new discoveries by elucidating the reasoning behind predictions and enforce *Right of Explanation* policies [2]. Explanations are reported by assigning relative importance to the input variables. Notably, different XAI methods adopt different approximations to determine this importance in relation to the ML model [3–7]. These distinct techniques come with their individual variances, underpinned by a foundation of internal model uncertainty that forms the basis for their explanations. As these XAI methods yield outcomes with varying degrees of uncertainty [8–10], it raises concerns about potential distrust among end-users. Thus, in this study, we delve into how the internal model representations affect the stability of these explanations, notably in the field of toxicity prediction.

Historically, computational toxicity prediction relied on statistical methods, structural alerts, and quantitative structure-activity relationship (QSAR) models [11], often based on chemical descriptors or fingerprint representations. However, a recent surge in popularity revolves around natural language processing (NLP) used for text-based representation learning in molecular modeling, exemplified by the high accuracy of the transformer CNN [12] for Ames mutagenicity prediction [13]. The Ames test [14] serves as a screen for mutagenic compounds, and had important advantages when it was first introduced, including less test chemical, time, plastic and can be automated [15]. In NLP-based molecular representation for prediction of Ames, Simplified Molecular Input Line Entry System (SMILES) [16] are used to represent molecules as a text string. These SMILES are then used within an NLP-driven machine learning (ML) model, such as sequence-to-sequence (seq2seq) [17, 18]. Later state-of-the-art NLP models used augmentation strategies including masking [19, 20], which also spread to NLP models for cheminformatics [12, 21, 22]. Furthermore, the high accuracy of the transformer CNN was partly achieved with data enumeration. Comparable to vision-based ML, where images are rotated to augment the training data, researchers used multiple SMILES strings representing the same molecule [23]. This was then used to optimize internal model representations in an auto-translation task to capture intricate structural nuances [24].

When model input can be directly translated to structural parts of a molecule, explanations from XAI methods more naturally correspond to human intuition. These XAI techniques extend their importance assessment capabilities across global and local dimensions. On the global context, the evaluation centers on the role of variables in shaping the model’s overall construction [25]. In the local context, the focus shifts to the influence of variables on specific predictions, a facet that aligns with our current investigation. Local prediction elucidation strategies have emerged in the literature, including approximating complex models with simpler ML counterparts [5], or leveraging game theory to pinpoint influential input variables by comparing a selection of subsets [4]. Alternatively, researchers have harnessed the model’s intrinsic architecture to unveil its decision-making processes. In deep learning, integrated gradients [3] use accumulate local gradients to assess variable effects on output. Alternatively, XAI can use inherently interpretive parts of a model. The latter has proven especially influential by using the attention mechanism of transformers, exemplified by recent work from Qiang et al. [7] that utilize the transformer attention mechanism thought to be inherently interpretable [18].

Contrasting multiple input parameters that represent the same underlying ground truth is a method to assess the stability of a model or method. This test-time data augmentation has been used in prior research conducted in domains including medical imaging, where data augmentation techniques, such as image rotation and contrast adjustment, have been used to measure model uncertainty [26, 27]. These strategies have demonstrated their efficacy in enhancing model performance, gauging model uncertainty, and increasing robustness. However, this methodology remains largely unexplored within the field of XAI concerning molecular representations, presenting an opportunity to assess the robustness of XAI methods.

In this study, we delve into the challenges presented by Langer et al. [28] who investigated that stakeholders of ML models desire XAI methods to produce human-understandable and consistent interpretations. We focus on the robustness of local XAI methods for molecular representations, specifically by comparing importance assigned to equivalent SMILES representations. Our study has broad implications for the XAI field, given the potential impact of varying importance assignments in toxicity prediction. Our contributions can be summarized as follows:

- We investigate the influence of molecular pre-training settings on the transfer learning on Ames mutagenicity.
- We assess the robustness of eight XAI methods given different representations of the same underlying molecule, using test-time augmentation.
- The robustness is assessed using different data distributions, training variations and from the perspective of model randomization.
- Additionally, test-time augmentation is investigated as a means of increasing consistency between XAI methods for the same model and interpretations of the same XAI method from different learned representations.
- Finally, we provide a global-level comparison of model attributions with expert-based structural alerts.

## Background

Here, we describe the mathematics behind the transformer-based, deep learning and model agnostic methods investigated in this paper. The methods utilize some common parameters and variables including the sequence length *S*, embedding dimension *E*, number of attention heads *h*, and head-specific embedding dimension $d = E/h$, number of layers *L*. Furthermore, we define $\textbf{x}$ as the tokenized input sequence, Ames prediction model $f(\textbf{x}) = y, y \in \mathbb {R}^{2}$. XAI attributions are given as $\phi \in \mathbb {R}^{S\times E}$, where *E* can equal *S* depending on the XAI method. Because the dimensions of the embedding of $\phi$ can be different based on method, we average over the embedding space to make $\phi _i = \frac{1}{n}\sum _{j=0}^n\phi _{i,j}$, which means that $\phi _i, i \in (1,..., S)$ is consistent between methods.

### Transformer-based interpretation

Methods that depend on the transformer architecture primarily make use of the attention mechanism. We here redefine the methods proposed by Qiang et al. [7]. Vaswani et al. [18] defined the multi-head attention $\alpha ^l_h \in \mathbb {R}^{h \times S \times dh}$ of layer $l \in (0,..., L)$ (Eq. 1). Later usage of $\alpha _{i,j}$ corresponds to the values averaged over all heads. Furthermore, the output of each layer in the encoder is denoted as $o^l \in \mathbb {R}^{S\times E}$ is defined in Eq. 2.

1 $$\begin{aligned} \alpha _{i,j}^l=\text {softmax} \left( \frac{\textbf{Q}\textbf{K}^T}{\sqrt{d}} \right) \end{aligned}$$

2 $$\begin{aligned} h_{i,j}^l=\text {concat}\left( \varvec{\alpha }_{i,j,1} \textbf{V},...,\varvec{\alpha }_{i,j,h}\textbf{V}\right) W^O \end{aligned}$$

In Eqs. 1, 2$(\textbf{Q},\textbf{K},\textbf{V}) \in \mathbb {R}^{h \times S \times d}$ represent the projection matrices of query, key and values respectively, and $\textbf{W}^O \in \mathbb {R}^{E \times E}$ represent model weight matrix of the projection in multi-head attention. Bias is left out in the definitions for simplicity.

Firstly, [29] proposed to use the raw attention directly as an explanation of the entire model (Eq. 3). Usually the last layer of the encoder is used in this approach. Alternatively, the raw attention by multiplying all raw attentions over all layers of the model can be aggregated to form one explanation, as defined in Eq. 4.

3 $$\begin{aligned} \phi _{i, AttentionMaps}= & {} \frac{1}{n}\sum _{j=0}^n\alpha _{i,j}^L \end{aligned}$$

4 $$\begin{aligned} \phi _{i, Rollout}= & {} \frac{1}{n}\sum _{j=0}^n \prod _{l=0}^L (\alpha ^l {\textbf{I}}_S)_{i,j} \end{aligned}$$

In Eq. 4, $\textbf{I}_S \in \mathbb {R}^{S \times S}$ is the identity matrix.

Qiang et al. [7] further expanded research by Selvaraju et al. [30] by using the class-activated gradients. Here, class-activated gradients are denoted using $\nabla _{\omega }y_c$ with $\omega$ being the position with respect to which the gradients are calculated and $y_c$ is the index of the output corresponding to the class of interest *c* (in our case toxic or non-toxic). This was then used in the interpretation method of the attention layers (Eq. 5). Firstly, similarly to the attention maps, by using the last layer or a specific layer to explain the entire model. Furthermore, [7] expanded this by summing over all layers and combining the gradients with the raw attention (Eq. 6).

5 $$\begin{aligned} \phi _{i, Grad}= & {} \frac{1}{n}\sum _{j=0}^n (\nabla _{[\alpha ,L]}y_c)_{i,j} \end{aligned}$$

6 $$\begin{aligned} \phi _{i, AttGrad}= & {} \frac{1}{n}\sum _{j=0}^n\left( \sum _{l=0}^L (\nabla _{[\alpha ,l]}y_c)_{i,j} \odot \alpha _{i,j}^l\right) \end{aligned}$$

In Eq. 6, $\odot$ represents the Hadamard product.

Finally, [7] have defined rules to use the outputs of the attention layers instead of the attention itself. Here, they combine the outputs *o* with the class-activated gradients with respect to *o*, $\nabla _{o,l}y_c$ (Eq. 7) and together with the attention (Eq. 8).

7 $$\begin{aligned} \phi _{i, CAT}= & {} \frac{1}{n}\sum _{j=0}^n\left( \sum _{l=0}^L (\nabla _{h,l}y_c)_{i,j} \odot h_{i,j}^l\right) \end{aligned}$$

8 $$\begin{aligned} \phi _{i, AttCAT}= & {} \frac{1}{n}\sum _{j=0}^n\left( \sum _{l=0}^L \alpha _{i,j}^l \odot (\nabla _{h,l}y_c]_{i,j}) \odot h_{i,j}^l\right) \end{aligned}$$

### Deep learning-based interpretation

Methods that are dependent on deep learning, but not specifically transformers-based, usually depend on the calculation of the gradients for their interpretation. The most straightforward way is to use the basic full gradients over the entire model. Integrated gradients (IG) contrasts gradients of a prediction iteratively with the gradients of a background sample by integrating over the differences between input parameters of the sample $\textbf{x}$ and the input parameters of the empty background $\bar{\textbf{x}}$ (Eq. 9).

9 $$\begin{aligned} \phi _{i, IG} = \frac{1}{n}\sum _{j=0}^n\left( (x_{i,j} - \bar{x}_{i,j}) \int _{\alpha =0}^1 \frac{\delta f (\bar{x}+\alpha (x-\bar{x}))}{\delta x_{i,j}} d \alpha \right) \end{aligned}$$

### Model agnostic interpretation

The model agnostic methods use either inherently interpretable approximations of models [5], or other methods that perturb the input and evaluate model output variation. One perturbation technique is using Shapely additive values (Eq. 10) [4] where game theory of parameter subsets are used to identify the relative importance of parameters.

10 $$\begin{aligned} \phi _{i, SHAP} = \sum _{ \textbf{S} \subseteq \textbf{F} - \{k\}} \frac{|\textbf{S}|!(|\textbf{F}| - | \textbf{S}| - 1)!}{|\textbf{F}|!} \left( f_{\textbf{S} \cup \{k\}}(x_{\textbf{S} \cup \{k\}})-f_{\textbf{S}}(x_{\textbf{S}})\right) , \end{aligned}$$

In Eq. 10$\textbf{F}$ is the set of all subsets, $\{k\}$ the variable of interest, $\textbf{S}$ the sets of all subsets without $\{k\}$, and $x_{\textbf{S}}$ and $x_{\textbf{S} \cup \{k\}}$ are the input with the parameters of subset $\textbf{S}$ and $\textbf{S} \cup \{k\}$ respectively.

## Methods

### Data collection and processing

All data was gathered from Therapeutic Data Commons (TDC) (version 0.4.0) [31] which provided standardized output for the ChEMBL database (version 29) [32, 33] and the Ames dataset [13] including standardized splitting. Both datasets were cleaned using RDKit (version 22.9.3) [34]. Datasets were processed to remove stereochemistry and salts; to correct invalid hybridization, conjugation, chirality, and valency; and to set correct chirality, aromaticity and chemical property flags. Corresponding canonical SMILES were then generated and duplicates were removed. Finally, datapoints that had overlap between Ames and ChEMBL were removed from the ChEMBL dataset. SMILES were tokenized based on the character representations of the string format and given both a beginning-of-sequence (BOS) and end-of-sequence (EOS) token together with optional padding (PAD) tokens to make sequences of equal length, according to the original paper from Karpov et al. [12]. Structural alerts were gathered from Kazius et al. [35] and generated using RDKit SMARTS (SMILES arbitrary target specification) representations. Identification of alerts in molecules was performed using RDKit substructure search.

### Model architecture and training

**Fig. 1:**
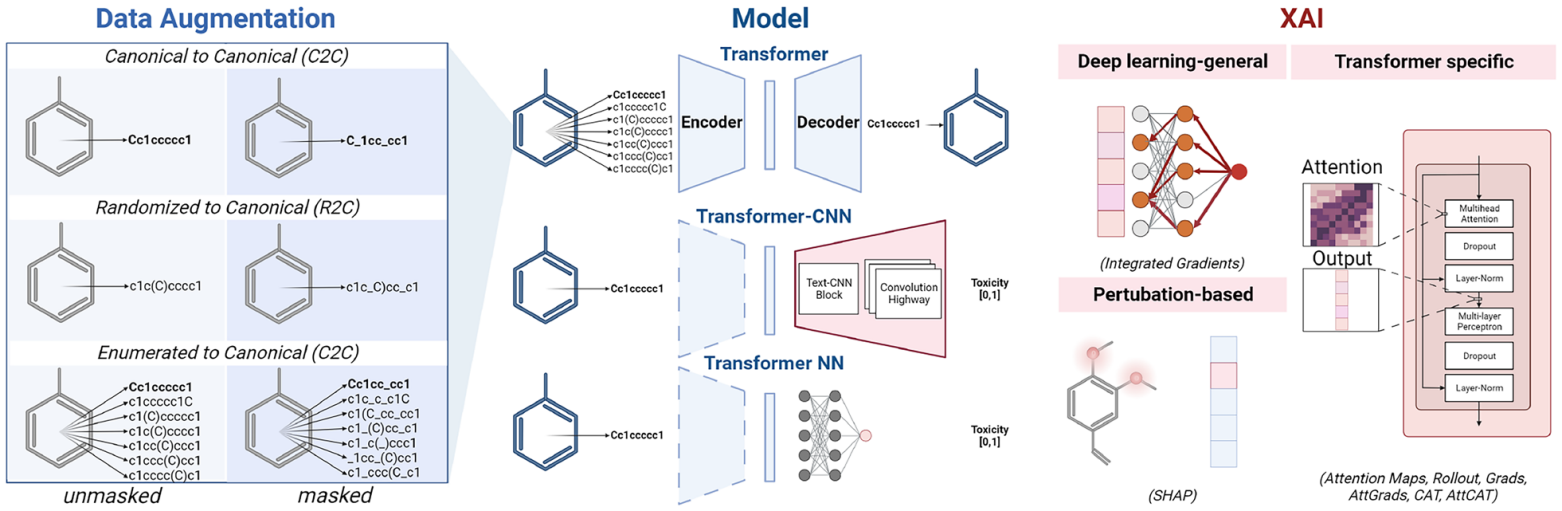
Overview of the methods used throughout the research. Data augmentation is used during pre-training. Transfer learning uses the pre-trained transformer encoder together with a small neural network or CNN. Thereafter, eight XAI methods subdivided into three groups were used for interpretation

Model training was performed using PyTorch (version 2.0.1) [36] and Lightning (version 2.0.5) [37]. Early stopping was implemented based on the validation cross entropy loss. Full model parameters for the training are described in Table 1 and a general overview of the methods used are visualized in Fig. 1.

**Table 1:** Model training details

	Training	
Parameter	Pre-training	Transfer learning
Batch size	128	128
Learning rate	$10^{-4}$	$5\times 10^{-5}$
Weight decay	0.01	0.01
Dropout	0.1	0.3
Initialization	Xavier	Xavier
Optimizer	AdamW	AdamW
Scheduler	None	None
Frozen encoder	No	Yes
Max sequence length	175	175
Token space	68	68
Embedding dimension	512	512

#### Representation learning

In this study, two transformer-based architectures were explored to learn the molecular representation of SMILES. The first is the encoder-decoder architecture that forms the basis of seq2seq [17] and BART [20] models. The second is the encoder-only architecture that formed the basis for the BERT model [19]. The sole difference between these architectures is that the decoder architecture in the former is a transformer block with cross attention and the latter is a simple multilayer perceptron.

The pre-training was based on the ChEMBL database (Table 2), where the task was auto translation. The architecture was trained to produce the canonical SMILES representation from either a canonical SMILES, randomized SMILES or an enumerated SMILES (up to ten randomized SMILES and one canonical SMILES). Additionally, masking was performed as another way to augment the training process, where 15% of the tokens in a SMILES string were replaced by 80% MASK tokens, 10% random character tokens and 10% unchanged tokens. Furthermore, alternative embedding dimensions and pruning options were also explored.

**Table 2:** ChEMBL data breakdown

	Size	Training	Validation	Test
ChEMBL 29	1.895.373	1.291.223	197.497	407.014
ChEMBL 29 (no Ames)	1.892.281	1.289.081	197.119	406.081

#### Ames transfer learning

The architectures were used for transfer learning by using the pre-trained encoders from the pre-trained models coupled with a small neural network, the predictor. Both the original implementation of the transformer CNN where the predictor was a TextCNN layer with a highway unit layer, as well as a simple max pooling layer with a multilayer perceptron were used as model variants for the transfer learning stage. Transfer learning was performed using canonical to a binary classification (toxic or non-toxic) (Table 3). During training, the pre-trained encoder were frozen to keep generalization capabilities, and trained on the scaffold split as provided by TDC. In order to give an indication of variance in performance whilst still only using the scaffold split, test-time bootstrapping was performed where the test set was sampled with replacement until full test length, and prediction statistics calculated 1000-fold to indicate variance (Tables 10, 11).

Additionally, we evaluated training variations, including transfer learning using enumerated SMILES to a binary classification (enumerated), training using a randomly initialized frozen encoder, a completely randomized model without further training and training using the random split instead of the scaffold split (Fig. 7). Details regarding data distribution differences between the data splits are found in Appendix A.

**Table 3:** Ames data breakdown

	Size	Scaffold split			Random split		
		Training	Validation	Test	Training	Validation	Test
Toxic		2511	373	753	2470	391	776
Non-toxic		2248	297	535	2139	297	644
Total	6717	4759	670	1288	4609	688	1420

### XAI methods

The XAI methods of IG and SHAP were used using captum [38]. All other methods were re-implemented. Methods are described in Table 4. For XAI methods that use a specific layer for their interpretation (Attention Maps, Grads), we used the last layer averaged over all attention heads. Furthermore, the original implementations of Grads and AttGrads, and CAT and AttCAT were re-implemented using the PyTorch [36] autograd system instead of hooks as in the original implementation [7].

**Table 4:** Descriptions and equations of metrics used during the analysis of the interpretations

Method	Implementation	Equations
IG	Captum	Eq. 9
SHAP	Captum	Eq. 10
Attention Maps	Re-implemented	Eq. 3
Rollout	Re-implemented	Eq. 4
Grads	Re-implemented	Eq. 5
AttGrads	Re-implemented	Eq. 6
CAT	Re-implemented	Eq. 7
AttCAT	Re-implemented	Eq. 8

Described are three metrics, what they measure and their equations. $\phi _i$ is the attribution of token *i*. Entropy and cosine similarity were calculated only using the atom components

### Statistical analysis

All attributions were obtained based on the SMILES sequences of molecules and represented each token attribution as $\phi _i$ with *i* corresponding to each token in the original full string. Analyses of token attributions were analysed in the normalized form were the original $\mathbf {\phi }$ attribution vector was divided by the absolute sum of the total string $\hat{\phi _i}=\frac{\phi _i}{||\mathbf {\phi }||}$. We understand $\mathbf {\phi }$ to correspond the attributions of the full tokenized string, including original SMILES string, BOS, EOS and PAD tokens. Other analyses include analyses of components of SMILES, atom and alerts (Table 5). As mentioned, all analysed components were first normalized with respect to the full tokenized string.

**Table 5:** Names of components, description of components and example of token strings

Name	Analysed components	Example
Full	Full token string	*BOS* O=C=O *EOS* *PAD* *PAD*
SMILES	Full SMILES string	O=C=O
Atom	Atom tokens ordered canonically	COO
Alerts	Atom tokens of structural alerts	CO

Described are four names with corresponding sections of the tokens analysed in statistical analyses. Most analyses use atom tokens or alerts but are relative to the full tokenized string

We investigated the normalized attributions by comparing distances between attributions, entropy within the attributions and the relative importance given to relative components of the distributions (Table 6). Cosine similarity was chosen as a distance measure to analyse the variation of importance over attributions using the implementation from SciPy [39]. Cosine similarity was chosen to measure the agreement of importance given to each of the tokens. Entropy was calculated as a measure of information, similar to Dabkowski and Gal [40], and relative importance is the fraction of the attribution given to a specific component of the input (Table 5), mostly to investigate the overlap between XAI information and human-derived structural alerts [35].

**Table 6:** Descriptions and equations of metrics used during the analysis of the interpretations

Metric	Analyses	Equation
Cosine distance	Distance between attributions	$cos(\theta ) = 1-\frac{u \cdot v}{||u||_2||v||_2}$.
Entropy	Information contained in the attribution	$H(\phi ) = - \sum _{i=0}^n \phi _i \times log_2(\phi _i)$
Relative importance	Relative importance given to component	$\sum \phi _i, \phi _i \in \text {component}$

Described are three metrics, what they measure and their equations. $\phi _i$ is the attribution of token *i*. Entropy and cosine similarity were calculated only using the atom components

### Computational efficiency

To avoid unnecessary computational overhead, all models were kept small 16.5 M parameters or lower, trained on the smaller ChEMBL dataset rather than the more standard PubChem dataset and using 16-bit precision. All models can be trained using a single GPU. The longest pre-training time in our experiments was 24 h (masked enumerated encoder-decoder) using two GPUs with distributed training for faster and more efficient computing.

### Reproducibility

The implementation code are publicly available on GitHub at https://github.com/PeterHartog/augmented-xai to ensure the reproducibility of the experiments. Cleaned data and trained model weights are publicly available from Figshare: https://doi.org/10.6084/m9.figshare.24866091.

## Results

### Model training

#### ChEMBL representation learning

To generate our internal representations, we first trained attention-based encoder and attention-based decoder (encoder-decoder) and attention-based encoder with an MLP (encoder-only) transformer models on the ChEMBL small molecule dataset (Table 8). Training regimens were to translate canonical to canonical (C2C), randomized to canonical (R2C) and enumerated to canonical (E2C) as well as the masked versions (e.g., ME2C). Additionally, the encoder-decoder models character and sequence accuracy scores for the canonical to canonical versions were high in all versions except for the R2C models. The encoder-decoder R2C models were more predictive regarding the sequence accuracy without context (greedy-search), but still significantly worse than other training regimens.

#### Ames transfer learning

To create our final prediction models, we used transfer learning of the pre-trained models to the Ames training set by freezing the encoders and replacing the decoders by either a textCNN or MLP. The MLP results on the scaffold split (Table 7) outperformed the transformerCNN model (Table 9). Because of this, we decided to continue our interpretation analysis with the MLP model. Pre-trained models of C2C and E2C outperformed R2C models, whilst masked models increased model statistics over unmasked models. Finally, encoder-only pre-trained models generally outperformed encoder-decoder models on the Ames transfer learning task.

**Table 7:** AUROC, accuracy, F1, MCC precision and recall scores of MLP models transfer learned on Ames data

	Training	AUROC$\uparrow$	Accuracy$\uparrow$	F1$\uparrow$	MCC$\uparrow$	Precision$\uparrow$	Recall$\uparrow$
No training	Untrained	0.652	0.516	0.143	0.063	0.081	0.619
	Native	0.676	0.634	0.624	0.269	0.607	0.642
Variations	Random split	0.856	0.788	0.788	0.576	0.789	0.787
	Train set	0.873	0.792	0.792	0.584	0.792	0.792
	CNN	0.709	0.650	0.657	0.300	0.671	0.644
	Enumerated	0.810	0.739	0.739	0.478	0.738	0.739
Encoder only	C2C	0.734	0.666	0.666	0.332	0.665	0.666
	R2C	0.738	0.670	0.670	0.339	0.670	0.670
	E2C	0.731	0.665	0.665	0.331	0.665	0.665
	MC2C	0.754	0.682	0.682	0.364	0.683	0.682
	MR2C	0.694	0.653	0.652	0.305	0.651	0.653
	ME2C	0.804	0.719	0.719	0.438	0.719	0.719
Encoder-decoder	C2C	0.716	0.662	0.662	0.324	0.663	0.662
	R2C	0.698	0.634	0.634	0.269	0.634	0.634
	E2C	0.748	0.677	0.678	0.354	0.679	0.676
	MC2C	0.751	0.682	0.682	0.365	0.681	0.683
	MR2C	0.698	0.647	0.647	0.294	0.647	0.647
	ME2C	0.772	0.696	0.697	0.393	0.699	0.695

Values are based on the scaffold split

### Interpretation analysis

**Fig. 2:**
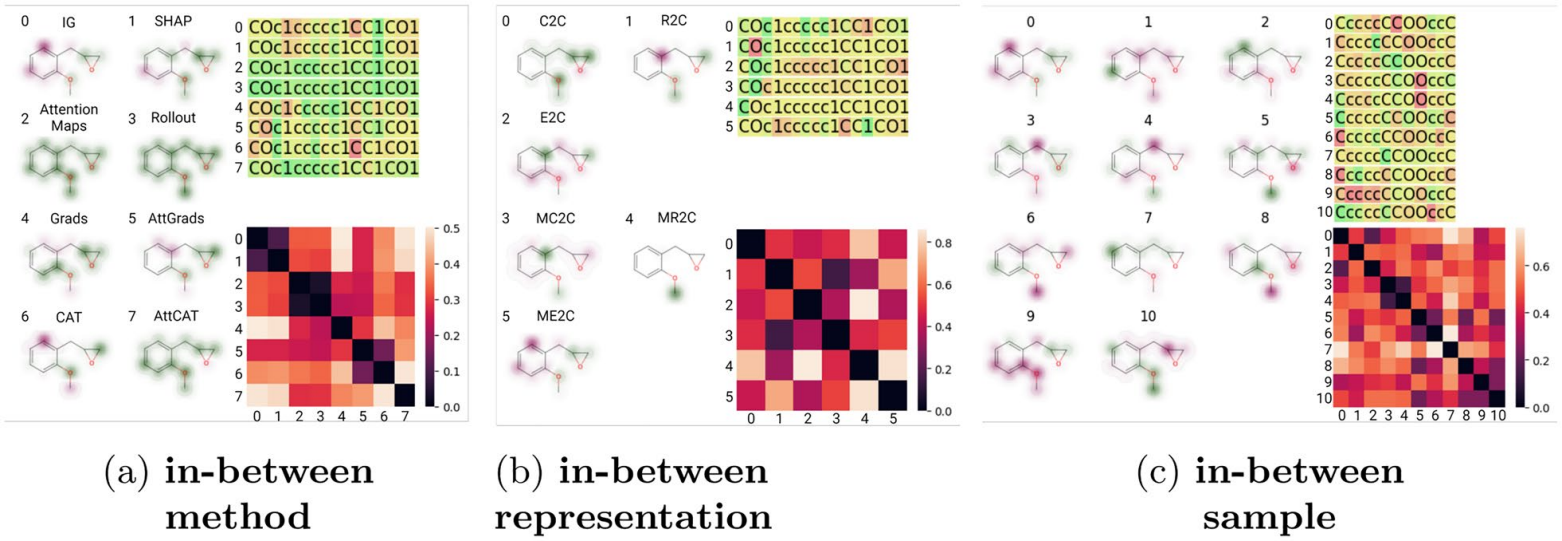
Single instance robustness analysis of XAI methods from three different angles: in-between method, in-between representation and in-between input. **a** Importance given to each input parameter varied over XAI methods, given a constant canonical SMILES input and encoder-decoder masked enumerated to canonical (ME2C) model. **b** Importance given to each input parameter varied over representations of the encoder-decoder model, given a constant canonical SMILES input and IG XAI method. **c** Importance given to each input parameter varied over different SMILES representations, given a constant canonical SMILES input, IG XAI method and encoder-decoder masked enumerated to canonical (ME2C) model

The robustness of XAI methods was examined from three different perspectives. The first was to analyse XAI methods in the same circumstance, namely the same model and the same input, to see how different the explanation given is over all methods. Figure 2a shows the different attributions from each XAI method for one randomly chosen canonical SMILES of the encoder-decoder ME2C model. Secondly, we want to investigate the variance that internal representation gives. To further illustrate this, the heat map shows the inner variance of the method, and the robustness score is the mean value of this heat map. Figure 2b shows how the different training regimens for an encoder-decoder architecture influence the explanations of IG for on randomly chosen canonical SMILES. Again, the heat map shows the internal consistency for in-between model robustness. Finally, we investigate how input variation all representing the same underlying ground truth, can change the given importance of IG for the encoder-decoder ME2C model. In Fig. 2c, we investigate the canonical SMILES and ten randomized SMILES and compare the internal robustness between the importance given to the atom indices. Importantly, we reshuffle the respective SMILES strings according to RDKit canonical atom order so that each character represents the same atom when compared.

#### Test-time augmentation as a measure for robustness

**Fig. 3:**
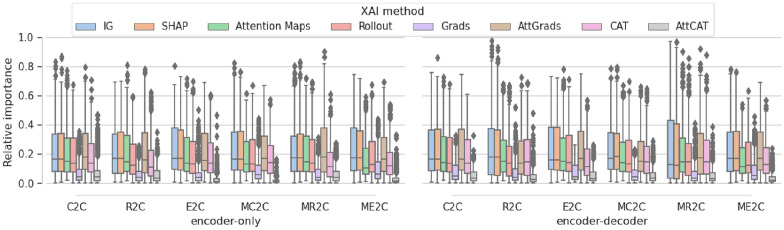
In-between sample distance robustness analysis of XAI methods. Cosine distances of attributions given to different SMILES representations of the same molecule over different XAI methods with different pre-trained representation models

Firstly, we analyse the difference between SMILES of the same molecules for each method and model training (Fig. 3). Overall, the cosine distance between these samples is greatest in the IG, SHAP and AttGrads and AttCat. Additionally, the distances of the attention maps, rollout attention are lowest and most variable. The Grads value seem to have the highest variability in most models. All other methods are relatively similar in all models, with the exception of the R2C and MR2C pre-trained models, where all methods have increased cosine distances in the encoder-decoder models, but less pronounced differences in the encoder-only models.

Additionally, we investigate whether the distances can be explained by the difference between canonical and random SMILES. No significant differences are found between distances of canonical and a randomly chosen random SMILES in most models. Some exceptions were found, as determined by the Mann Whitney U test (Table 12): CAT and AttCAT for encoder-only ME2C; encoder-decoder C2C AttGrads; encoder-decoder R2C Rollout and AttGrads; encoder-decoder E2C Attention Maps; encoder-decoder MC2C IG and SHAP. In these models the populations do look similar (Fig. 8), but results of these method-model combinations could possibly be explained by randomization attribution differences.

Finally, we utilize entropy as a measure for information to examine if the in-between sample analysis was due to overall small differences between values (Fig. 9). Entropy scores of IG, SHAP, Attention Maps, Rollout attention, AttGrad and CAT were similarly high throughout all models, but the values for Grads and AttCAT were significantly lower, indicating that the test-time augmentation robustness is partially dependent on the entropy the method itself.

#### Influence of ML model on XAI methods

**Fig. 4:**
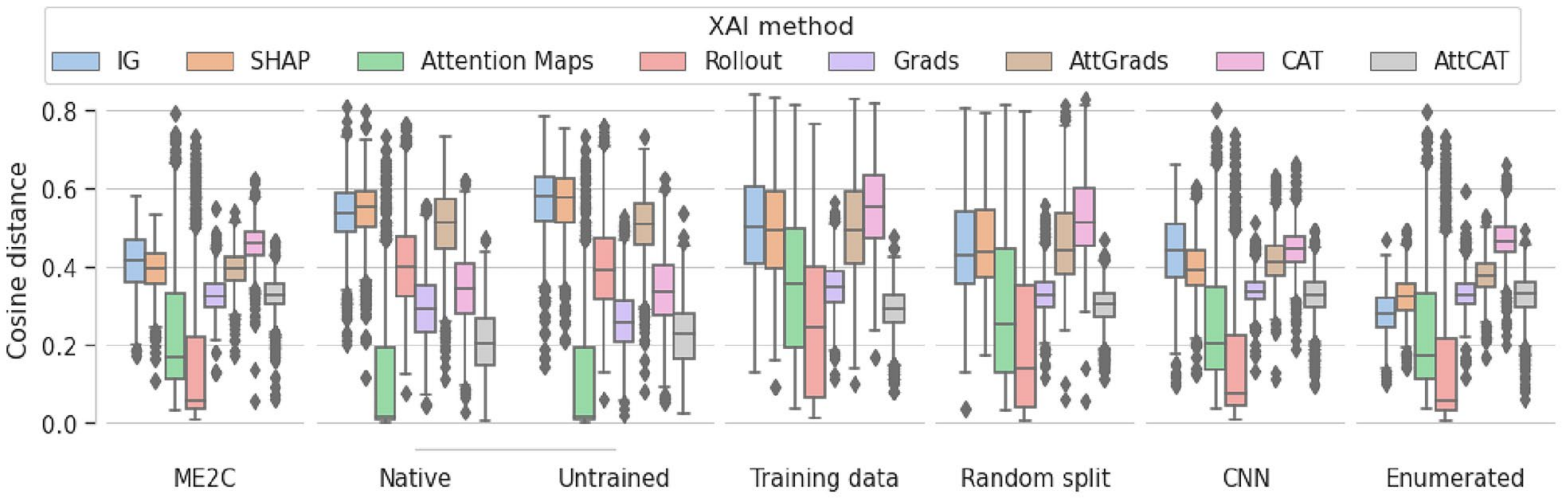
In-between sample cosine distance of different training settings. Cosine distances of different attributions given to different SMILES representations per molecule of different XAI methods of different training settings. Training settings include baseline ME2C of the encoder-decoder pre-training architecture, untrained, frozen encoder (native), completely random model (untrained), distances of the training data (train) of baseline, distances of the test set on a random split model (random), enumerated training (enumerated) and the statistics of the CNN model (CNN). All variations had the ME2C encoder-decoder model as the initial encoder with the exception of native and untrained

To investigate the dependence of XAI on models, we investigate the difference of both our test-time augmentation robustness score and the overall entropy of the XAI methods between different models. Firstly, differences between the pre-trained model with AUROC of 0.798, native model with AUROC of 0.763 and untrained model with AUROC of 0.474 in both entropy and in-between sample variation are minimal (Fig. 4). This indicates that the in-between model analysis of these models is dependent on something other than learned parameters.

Secondly, we identify the difference between the in-domain training data and the out-of-domain test data, as well as in-domain test data from a model trained using a random split ((Fig. 4). The in-domain data shows a larger difference in in-between sample distance, indicating that the XAI methods depend more on learned parameters than the out-of-domain samples. It also indicates that larger cosine distances are not necessarily less consistent with model predictions. Finally, we also analyse if different training techniques (enumerated) or architecture (CNN) affects cosine distances. No obvious distance changes were found (Fig. 4).

Interestingly, investigations into differences in entropy (Fig. 10) based on these variations were minor. We also further investigated if the number of tokens explained the consistency in in-between sample cosine distances (Fig. 11), where it seemed to be carbon-dependent and explains the variation.

#### Using test-time augmentation to improve XAI robustness

**Fig. 5:**
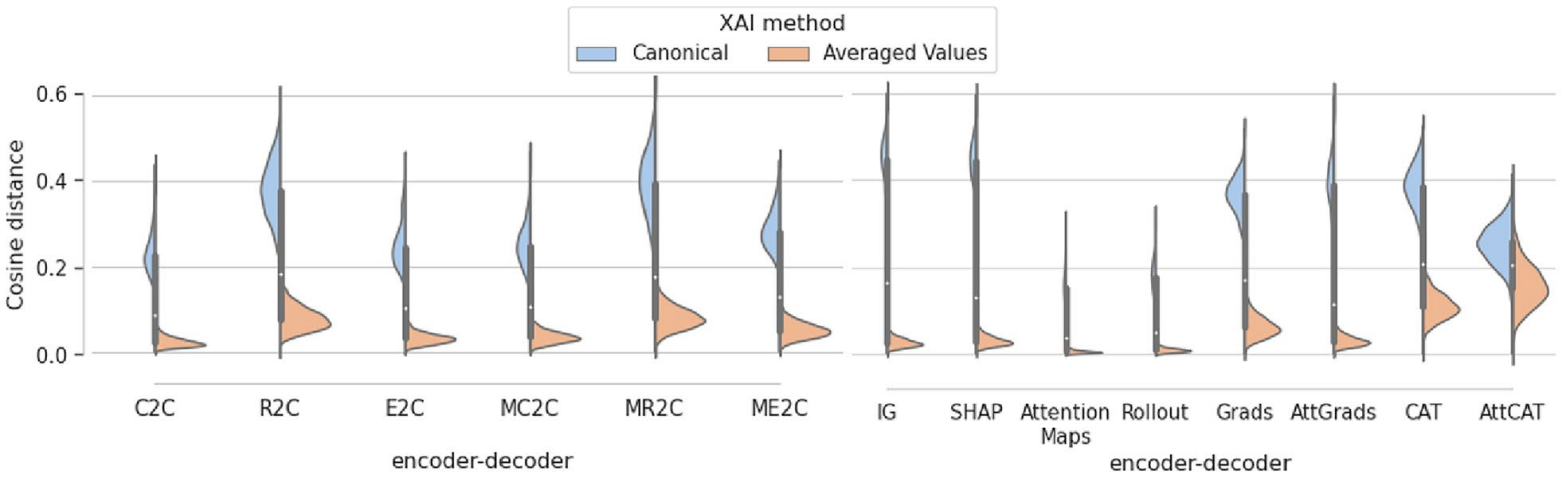
Comparison of the canonical and averaged values of in-between model and in-between method cosine distances. Cosine distances between different encoder-decoder models for the same method (in-between model) and different methods for the same encoder-decoder model (in-between method) of canonical and averaged atom attributions

To further assess the use of test-time augmentation, we analysed the in-between model and in-between method distances (Fig. 5). Figure 5 indicates that both the in-between model distance and the in-between method distances are reduced when values are averaged using test-time augmentation. This means that values become more consistent when using the average over test-time augmentation between both methods and models. We further investigated whether this effect was because of consistency or overall reduction in information by investigating the entropy of averaged values and canonical values (Fig. 10). There was no difference found between averaged values or canonical values, indicating only consistency between methods change, not specific attributions.

#### Comparison with expert-based structural alerts

**Fig. 6:**
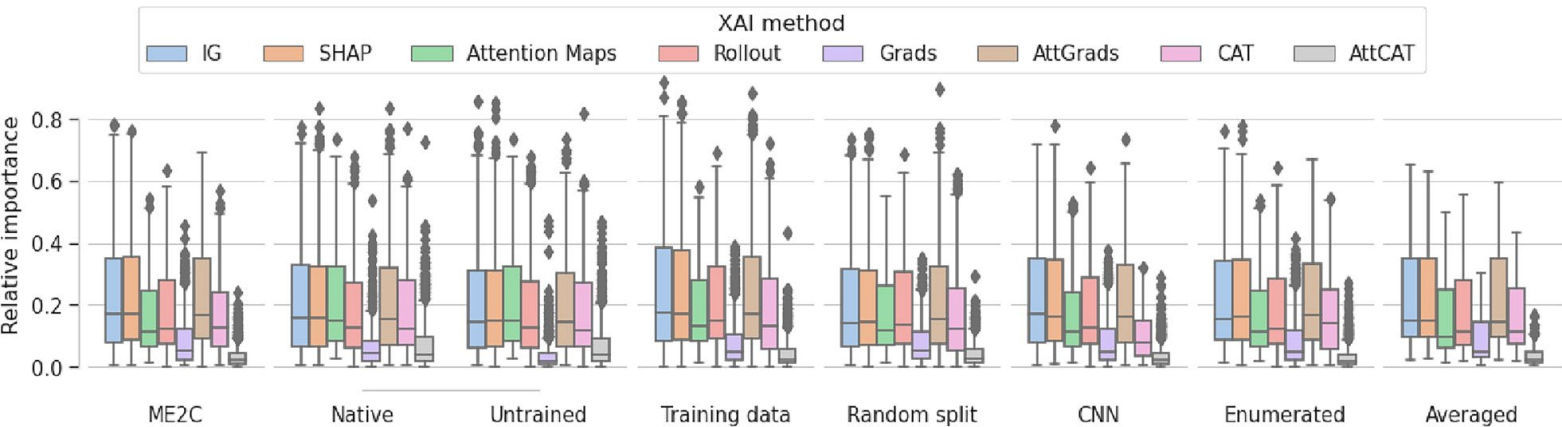
Relative importance given to expert-derived structural alerts. Relative token importance given to atoms corresponding to expert-derived structural alerts. Relative importance is given for canonical representations of different XAI methods of different training settings. Training settings include untrained, frozen encoder (native), completely random model (untrained), distances of the training data (train), distances of the test set on a random split model (random), enumerated training (enumerated), the statistics of the CNN model (CNN) and the averaged values of all SMILES enumerations of the ME2C model

**Fig. 7:**
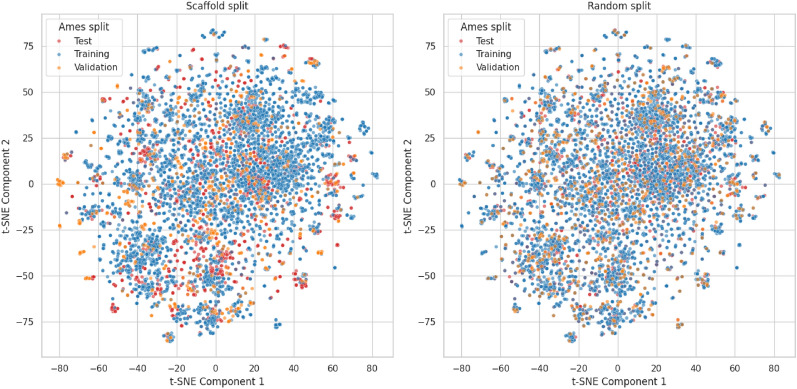
t-SNE plots of the distributions of training, validation and test for different splits of the Ames data set

**Fig. 8:**
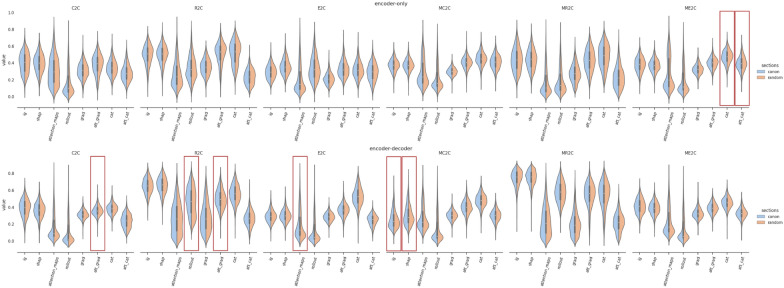
Violin plot of in-sample cosine distance populations per model and XAI method. Red boxes indicate p > 0.005 and notes that the populations are not significantly similar

**Fig. 9:**
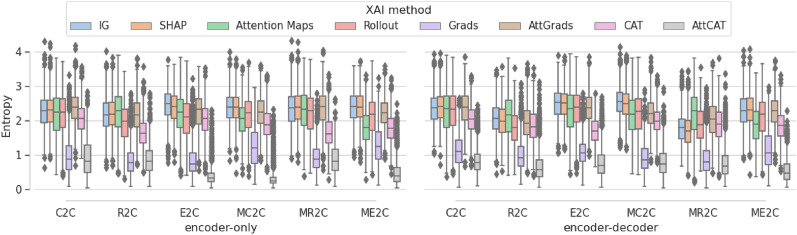
Entropy values of each XAI method per model. Entropy values of XAI methods of canonical representations indicating relative information in the attributions

**Fig. 10:**
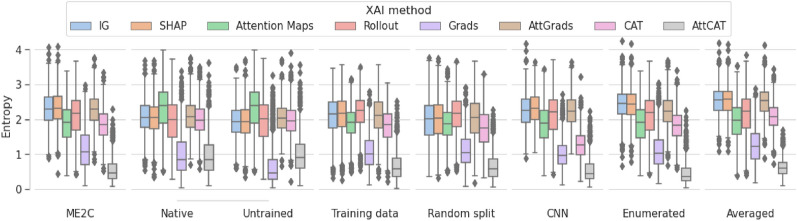
Entropy values of each XAI method per experiment. Entropy values of XAI methods of canonical representations or indicating relative information in the attributions. Averaged experiment variation used the average over all enumerations instead of the canonical representation for it’s entropy analysis

Finally, we analyse the robustness of model explanation by examining the overall attribution of all tokens as opposed to tokens corresponding to expert-derived structural alerts (Fig. 6). Values of IG, SHAP and AttGrads all consistently had the highest values with mean values around 0.2, whereas a Attention Maps, Rollout and CAT gave consistently similar or slightly lower relative importance. Relative importance of Grads and AttCAT consistently gave the lowest relative attribution to the structural alerts. Relative importance is generally consistent between randomized models, in-domain samples, training variations and test-time augmentation averaging. Similar consistent results were observed in model variations (Fig. 12).

We further analysed the overall attribution of all tokens as opposed to the SMILES tokens, atom tokens and tokens corresponding to expert-derived structural alerts of all methods for each model (Fig. 14) and all methods for each variation (Fig. 13), where similar attributions were found irregardless of model or variation.

## Discussion

In this research, we performed an analysis of eight XAI methods and used (1) test-time augmentation, (2) in-between model, method and sample cosine distances, (3) entropy, and (4) relative importance given to structural alerts to assess the validity of these XAI methods and XAI robustness analyses in the context of NLP-based molecular-representation models for toxicity.

### The importance of tokenization in NLP-based research

**Fig. 11:**
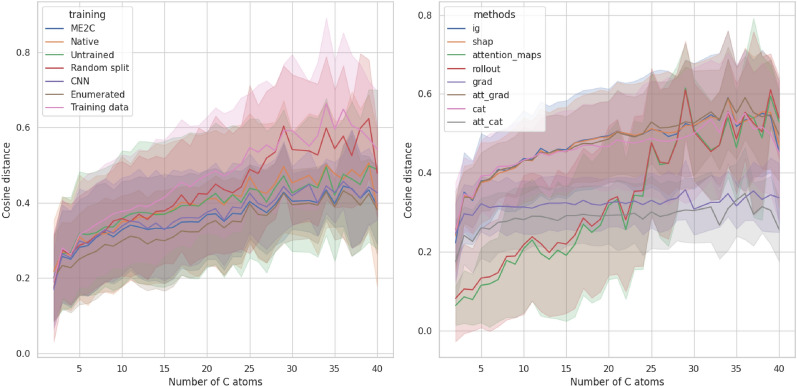
Cosine distance as a measure of number of carbon atoms. Variations of in-between sample cosine distances and carbon atoms, broken down to show training variations and XAI methods

**Fig. 12:**
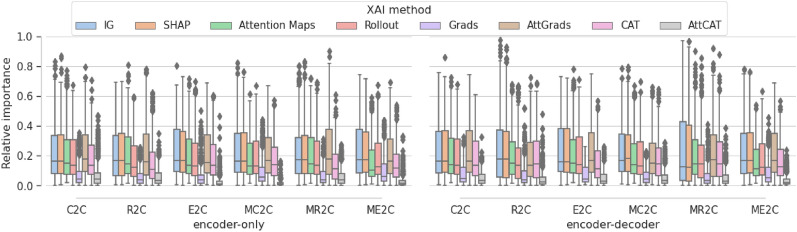
Relative importance given to expert-derived structural alerts for models. Relative token importance given to atoms corresponding to expert-derived structural alerts. Relative importance is given for canonical representations of different XAI methods of different pre-trained representation models

**Fig. 13:**
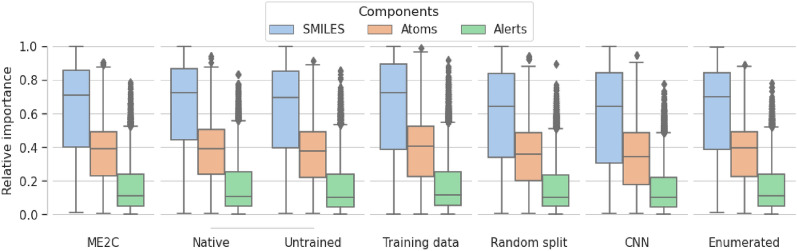
Relative importance given to different components for experiments. Relative token importance given to smiles, atoms and atoms corresponding to expert-derived structural alerts. Relative importance is given for canonical representations and aggregated over all XAI methods of different training settings

**Fig. 14:**
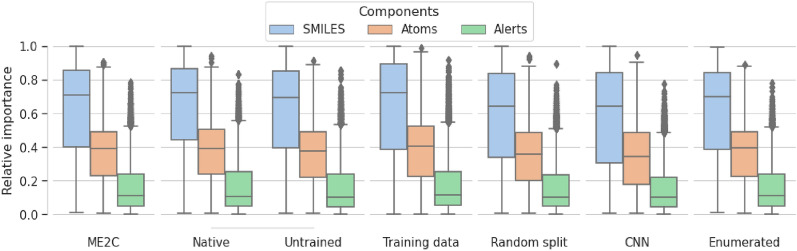
Relative importance given to different components for experiments. Relative token importance given to smiles, atoms and atoms corresponding to expert-derived structural alerts. Relative importance is given for canonical representations and aggregated over all XAI methods of different pre-trained representation models

Notably, we show that overall cosine distances between samples of a trained model and progressively randomly initiated models is minimal. This indicates that model interpretation in these cases is dependent on an aspect other than the learned model parameters. We analyse the variation with respect to the amount of carbon atoms in Fig. 11, which shows that consistency is dependent on the amount of carbon atoms. This indicates that tokenization is what is measured mostly by these interpretation methods. In fact, we find this aligns with the previous research of Zafar et al. [41] who found that interpretation of untrained models is not random in NLP-based models.

We hypothesise that the questions about the intuitive attributions of random models posed by Zafar et al. [41] can be answered by assessing the influence of the initial tokenization or architecture artifacts. This is further supported by our findings that a native encoder sometimes even outperformed pre-trained encoders during transfer learning, as well as research from Ucak et al. [42] who showed that different tokenizations can have significant effects on final performance in NLP-based molecular representation models.

### Usage of human intuition to validate XAI

In this study, we also used expert-derived structural alerts for Ames mutagenicity to investigate if the XAI overlapped with human intuition. A recent study investigated the mean attention weights given to toxicophores in Tox21 prediction and found significantly higher attention weights to toxicophore atoms than non-toxicophore atoms [43]. In this study, we analyse the overall importance given to structural alerts and don’t divide by the number of atoms. Regardless, in this study we also show that the relative importance given to structural alerts is mostly model-independent and, crucially, no different from completely randomized models. Structural alerts and case studies are often used to investigate the inner workings of ML research, but this finding urges caution using these tactics without further investigation.

### Test-time augmentation to improve XAI

Recently, [44] independently analysed the invariance and equivariance of interpretability methods. They used the bag-of-words [45] NLP model in combination with text permutation to assess the robustness of a number of feature importance methods, including IG and gradient SHAP [46]. Bag-of-words models differ from our models in that text data in bag-of-words is inherently invariant to text permutation, whereas different text representations in our approach are learned to be invariant given the same underlying molecule. Crabbé and van der Schaar postulated that “...Any interpretability method can be made invariant..., one should increase the number of samples $N_{inv}$ until the desired invariance is achieved. In this way, the method is made robust without increasing the number of calls more than necessary” [44]. This is consistent with our findings using test-time augmentation to improve XAI robustness, where we see greater in-between model and in-between method consistency when using values averaged over multiple $N_{inv}$ samples (i.e., test-time augmentations). This was even true in a model that was not inherently invariant.

However, we also identified that neither the amount of information analysed through entropy, nor the relative importance given to expert-derived structural alerts changes when using averaged values. This indicates that test-time augmentation can be used to make XAI more invariant, but not to improve XAI attribution overall.

### XAI methods and test-time augmentation for out-of-domain identification

Wang et al. [26] analysed test-time augmentation as a measure of aleatoric (data-based) uncertainty in the task of image segmentation and found it improved over baseline methods. Later, [27] used test-time augmentation to measure epistemic (model-based) uncertainty in the field of image classification where it again found to improve uncertainty classification. Uncertainty can be useful to identify out-of-domain predictions, but, to our knowledge, test-time augmentation has not yet been applied to that area of uncertainty prediction.

In this research, we hypothesize that test-time augmentation has a difference between in-domain and out-of-domain predictions. It remains to be seen if these differences can be used to determine out-of-domain predictions, especially in the field of NLP-based molecular representations. This is because we find less variation in out-of-domain in-between sample interpretations than the variation of in-domain interpretations. This indicates that if the epistemic uncertainty is measures the same model effects as XAI methods described here, it will show decreased values in uncertainty for out-of-domain samples. Additionally, this result identifies the need to combine XAI explanations with applicability domain assessments, to verify the explanation.

### Test-time augmentation as a measure for robustness

In this study, we analysed test-time augmentation as a measure for XAI robustness. We show substantial disagreement between augmented SMILES, even when canonical SMILES show no more difference than randomized SMILES. However, we also note a number of cautionary findings, including similar disagreements in randomized models, higher disagreement in in-domain distributions and relatively consistent distribution rankings. We did observe higher variation in in-between sample distances of untrained and worse-performing models (R2C and MR2C). This finding was subsequently diminished by the finding that in-domain training set values and random split values had similarly increased variation and higher overall values of in-between sample distances. We therefore conclude that using test-time augmentation as a measure for XAI robustness is inherently valid, but requires a comparison to randomized and applicability domain to draw well-founded conclusions.

### XAI implementation differences

Overall, we have investigated eight XAI methods, of which six are transformer-specific, one more general to neural networks and one perturbation XAI method. Firstly, we note that all methods from Qiang et al. [7] were re-implemented, and crucially, the methods of Grads, AttGrads, CAT and AttCAT were re-implemented with changes to the original implementation. Namely, for the gradients of the attention ($\nabla \alpha _{i,j}$) and the attention output ($\nabla h_{i,j}$), we implemented the gradients with respect to the full outcome. We believe this to be in line with the original publication, but have not tested differences due to implementation difficulties. Due to this, investigations into Grads, AttGrads, CAT and AttCAT were can be subject to change based on implementation. We did find the methods of Grads and AttCAT to have significantly lower entropy and lower relative importance to the structural alerts. However, the difference with respect to gradient calculations should not impact our final conclusions, as the methods still use the same underlying principles.

Furthermore, the methods of attention maps and Grads were performed using the last layer and averaged over all attention heads. Some researchers have suggested to use specific layer and head combinations to explain the model based on heuristic approaches, such as Schwaller et al. [47]. We leave such analyses to future papers and stay consistent here with the methods from Qiang et al. [7]. The methods of IG and SHAP were implemented using the standard Captum [38] library and can therefore serve as proper baseline implementations.

### Future investigation of XAI methods for SMILES-based representation models

In general, our findings indicate a greater need to identify what XAI methods measure, and specifically to remove any confounding background information. In our case, all methods in this context seemed to rely not on learned gradients, but likely instead on the tokenization. While this could be the ground truth explanation, randomization experiments indicated that this is not the case. We therefore advocate for approaches that properly take background into account. Notably, local XAI methods are often further refined through comparative analysis, contrasting their findings with those obtained from background or empty samples. Two of our methods included such a comparison to a background sample, namely SHAP and IG. The approach contrasted the input with padding tokens. This did increase their in-between sample distances in randomization studies, but this effect did not translate to the in-domain predictions, which saw similar distances albeit with higher variation. Interestingly, these methods were the only ones affected in the training variations of enumerated training on Ames and the CNN architecture, where other methods stayed consistent.

However, further comparisons, specifically with regards to randomization should help improve robustness in XAI methods. Although, as [8] discussed, randomization can indicate architecture-based priors of XAI, XAI should reflect learned parameters to explain model behaviour. Methods to increase XAI robustness to randomized models can include but are not limited to creating models to investigate ML models, such as [10], contrasting findings to randomized models predictions as background instead of empty samples, and counterfactual explanations, such as the recent study from Fradkin et al. [48].

Finally, we hypothesize that test-time augmentation can improve XAI methods and identify robust XAI methods, but only when XAI methods are both in-domain and measure decision-dependent parameters, not confounding information.

## Conclusion

In this research, we performed an analysis of eight XAI methods and used several analyses to assess the validity of these XAI methods and XAI robustness in the context of NLP-based molecular-representation models for toxicity. We report significant differences between explanations for different representations of the same ground truth. Additionally, we show that randomized models are similarly different, indicating that the XAI methods applied to NLP-based molecular representations in this and past research reflect tokenization more than learned parameters. Interestingly, we see a greater variance between in-domain predictions than out-of-domain predictions, further supporting this hypothesis. Furthermore, we investigated the relative importance given to expert-derived structural alerts and find similar importance given irregardless of applicability domain, randomization and training variation. We therefore caution future research to validate their methods using a similar comparison to human intuition without further investigation into the validity and robustness of the XAI method used. Finally, we note that test-time augmentation can be used as a measure of robustness, only if used in conjunction with other XAI method analyses, and note a greater need to identify what XAI precisely measure before drawing conclusions.

## Data Availability

The cleaned ChEMBL and Ames files, as well as model parameters are available from Figshare: https://doi.org/10.6084/m9.figshare.24866091.

## Appendix A

**Ames data analysis**

See Fig. 7.

## Appendix B

**Model statistics**

See Tables 8, 9.

**Table 8:** Model Statistics of pretrained transformer models

Architecture	Training	Canonical				Enumerated				Time (hh:mm)	Size
		Character accuracy		Sequence accuracy		Character accuracy		Sequence accuracy			
Encoder only	C2C	**1.000**		**1.000**		**1.000**		**1.000**		01:16	6.7M
	R2C	**0.202**		**0.000**		**0.195**		**0.000**		00:42	6.7M
	E2C	**1.000**		**1.000**		**1.000**		**1.000**		03:33	6.7M
	MC2C	**0.999**		**0.993**		**0.994**		**0.819**		01:25	6.7M
	MR2C	**0.202**		**0.000**		**0.196**		**0.000**		00:46	6.7M
	ME2C	**1.000**		**0.991**		**0.999**		**0.962**		10:31	6.7M
Encoder-decoder	C2C	0.998	**0.995**	0.994	**0.994**	0.998	**0.996**	0.994	**0.994**	01:45	15.9M
	R2C	0.813	**0.089**	0.000	**0.000**	0.554	**0.082**	0.000	**0.000**	02:44	15.9M
	E2C	0.998	**0.995**	0.995	**0.995**	0.998	**0.995**	0.994	**0.994**	08:43	15.9M
	MC2C	0.998	**0.996**	0.993	**0.987**	0.976	**0.965**	0.463	**0.452**	02:53	15.9M
	MR2C	0.812	**0.040**	0.000	**0.000**	0.549	**0.040**	0.000	**0.000**	05:56	15.9M
	ME2C	0.998	**0.994**	0.983	**0.958**	0.998	**0.994**	0.979	**0.936**	24:04	15.9M

Bold values are accuracies calculated without previous token information. Models were tested on canonical SMILES to canonical SMILES (canonical) and enumerated SMILES to canonical SMILES (enumerated)

**Table 9:** AUROC, accuracy, F1, MCC precision and recall scores of the TransformerCNN models transfer learned on Ames data

Architecture	Training	AUROC$\uparrow$	Accuracy$\uparrow$	F1$\uparrow$	MCC$\uparrow$	Precision$\uparrow$	Recall$\uparrow$
No training	Untrained	0.564	0.507	0.668	0.055	0.991	0.504
	Native	0.698	0.653	0.665	0.308	0.689	0.643
Encoder only	C2C	0.687	0.639	0.638	0.279	0.635	0.641
	R2C	0.706	0.643	0.652	0.287	0.668	0.636
	E2C	0.685	0.644	0.656	0.288	0.679	0.634
	MC2C	0.711	0.656	0.658	0.311	0.663	0.653
	MR2C	0.693	0.641	0.647	0.283	0.656	0.637
	ME2C	0.738	0.676	0.681	0.352	0.692	0.670
Encoder-decoder	C2C	0.711	0.663	0.662	0.327	0.658	0.665
	R2C	0.680	0.625	0.623	0.249	0.621	0.626
	E2C	0.719	0.650	0.650	0.301	0.649	0.651
	MC2C	0.715	0.643	0.647	0.287	0.655	0.640
	MR2C	0.652	0.583	0.583	0.167	0.582	0.583
	ME2C	0.709	0.650	0.657	0.300	0.671	0.644

Values are based on the scaffold split

## Appendix C

**Bootstrapping statistics**

See Tables 10, 11

**Table 10:** AUROC, accuracy, F1, MCC precision and recall scores with bootstrap variability of MLP models transfer learned on Ames data

Architecture	Training	AUROC$\uparrow$	Accuracy$\uparrow$	F1$\uparrow$	MCC$\uparrow$	Precision$\uparrow$	Recall$\uparrow$
No training	Untrained	0.640 ± 0.004	0.518 ± 0.003	0.172 ± 0.008	0.066 ± 0.011	0.100 ± 0.005	0.610 ± 0.019
	Native	0.652 ± 0.005	0.611 ± 0.006	0.601 ± 0.006	0.221 ± 0.012	0.587 ± 0.006	0.616 ± 0.006
Variations	Random split	0.853 ± 0.004	0.776 ± 0.006	0.776 ± 0.006	0.551 ± 0.012	0.776 ± 0.006	0.775 ± 0.006
	Training set	0.872 ± 0.002	0.789 ± 0.003	0.789 ± 0.003	0.577 ± 0.007	0.789 ± 0.003	0.788 ± 0.003
	CNN	0.721 ± 0.006	0.663 ± 0.008	0.668 ± 0.008	0.326 ± 0.016	0.678 ± 0.008	0.658 ± 0.008
	Enumerated	0.812 ± 0.003	0.740 ± 0.005	0.740 ± 0.005	0.480 ± 0.011	0.739 ± 0.005	0.741 ± 0.005
Encoder only	C2C	0.735 ± 0.004	0.666 ± 0.006	0.665 ± 0.006	0.332 ± 0.011	0.665 ± 0.006	0.666 ± 0.006
	R2C	0.735 ± 0.004	0.672 ± 0.006	0.672 ± 0.005	0.344 ± 0.011	0.672 ± 0.006	0.672 ± 0.006
	E2C	0.733 ± 0.004	0.657 ± 0.005	0.657 ± 0.005	0.315 ± 0.011	0.657 ± 0.005	0.658 ± 0.005
	MC2C	0.764 ± 0.004	0.695 ± 0.006	0.696 ± 0.006	0.389 ± 0.012	0.698 ± 0.006	0.693 ± 0.006
	MR2C	0.694 ± 0.003	0.648 ± 0.004	0.647 ± 0.004	0.296 ± 0.009	0.644 ± 0.004	0.649 ± 0.004
	ME2C	0.808 ± 0.003	0.734 ± 0.006	0.734 ± 0.006	0.468 ± 0.012	0.733 ± 0.006	0.735 ± 0.006
Encoder-decoder	C2C	0.715 ± 0.002	0.660 ± 0.005	0.661 ± 0.005	0.321 ± 0.009	0.663 ± 0.005	0.660 ± 0.005
	R2C	0.699 ± 0.005	0.640 ± 0.007	0.640 ± 0.007	0.281 ± 0.013	0.640 ± 0.007	0.640 ± 0.007
	E2C	0.748 ± 0.003	0.677 ± 0.006	0.678 ± 0.005	0.355 ± 0.011	0.680 ± 0.006	0.677 ± 0.006
	MC2C	0.748 ± 0.004	0.683 ± 0.006	0.682 ± 0.006	0.366 ± 0.012	0.681 ± 0.006	0.684 ± 0.006
	MR2C	0.687 ± 0.006	0.633 ± 0.007	0.633 ± 0.007	0.267 ± 0.013	0.633 ± 0.007	0.634 ± 0.007
	ME2C	0.782 ± 0.005	0.710 ± 0.007	0.710 ± 0.007	0.420 ± 0.014	0.711 ± 0.007	0.709 ± 0.007

Values are based on the scaffold split. ± values have been determined using 1000 fold test-time bootstrapping

**Table 11:** AUROC, accuracy, F1, MCC precision and recall scores with bootstrap variability of the TransformerCNN models transfer learned on Ames data

Architecture	Training	AUROC$\uparrow$	Accuracy$\uparrow$	F1$\uparrow$	MCC$\uparrow$	Precision$\uparrow$	Recall$\uparrow$
No training	Untrained	0.565 ± 0.006	0.502 ± 0.002	0.665 ± 0.001	0.019 ± 0.016	0.990 ± 0.002	0.501 ± 0.001
	Native	0.702 ± 0.005	0.659 ± 0.006	0.669 ± 0.006	0.318 ± 0.012	0.689 ± 0.007	0.650 ± 0.006
Encoder only	C2C	0.697 ± 0.005	0.654 ± 0.007	0.654 ± 0.007	0.309 ± 0.013	0.652 ± 0.007	0.655 ± 0.007
	R2C	0.714 ± 0.005	0.652 ± 0.007	0.660 ± 0.007	0.304 ± 0.013	0.676 ± 0.007	0.645 ± 0.006
	E2C	0.692 ± 0.005	0.649 ± 0.007	0.660 ± 0.007	0.298 ± 0.014	0.683 ± 0.007	0.639 ± 0.007
	MC2C	0.725 ± 0.006	0.660 ± 0.008	0.663 ± 0.008	0.320 ± 0.015	0.668 ± 0.008	0.657 ± 0.008
	MR2C	0.695 ± 0.005	0.643 ± 0.007	0.649 ± 0.007	0.287 ± 0.014	0.660 ± 0.007	0.638 ± 0.007
	ME2C	0.740 ± 0.006	0.680 ± 0.008	0.686 ± 0.007	0.360 ± 0.015	0.701 ± 0.008	0.673 ± 0.007
Encoder-decoder	C2C	0.711 ± 0.004	0.659 ± 0.006	0.657 ± 0.006	0.319 ± 0.012	0.652 ± 0.007	0.662 ± 0.006
	R2C	0.680 ± 0.006	0.634 ± 0.007	0.633 ± 0.008	0.269 ± 0.015	0.631 ± 0.008	0.635 ± 0.008
	E2C	0.713 ± 0.004	0.653 ± 0.006	0.652 ± 0.006	0.306 ± 0.012	0.649 ± 0.006	0.655 ± 0.006
	MC2C	0.726 ± 0.006	0.663 ± 0.008	0.667 ± 0.007	0.326 ± 0.015	0.676 ± 0.008	0.659 ± 0.007
	MR2C	0.644 ± 0.004	0.584 ± 0.001	0.583 ± 0.001	0.167 ± 0.001	0.583 ± 0.001	0.584 ± 0.001
	ME2C	0.721 ± 0.006	0.663 ± 0.008	0.668 ± 0.008	0.326 ± 0.016	0.678 ± 0.008	0.658 ± 0.008

Values are based on the scaffold split. ± values have been determined using 1000 fold test-time bootstrapping

## Appendix D

**Differences between canonical and randomized smiles distances**

See Fig. 8 and Table 12

**Table 12:** Mann Whitney U test statistics of the difference in in-sample distance populations for each model and interpretation method

Architecture	Training	IG	SHAP	Att.	Rollout	Grads	AttGrads	CAT	AttCAT
				Maps					
Encoder only	C2C	0.761	0.867	0.767	0.722	0.831	0.032	0.930	0.673
	R2C	0.121	0.114	0.507	0.339	0.316	0.036	0.376	0.469
	E2C	0.375	0.796	0.871	0.299	0.667	0.908	0.900	0.081
	MC2C	0.848	0.978	0.370	0.548	0.281	0.342	0.921	0.409
	MR2C	0.711	0.760	0.925	0.793	0.903	0.278	0.457	0.855
	ME2C	0.216	0.373	0.626	0.955	0.326	0.060	**0.004**	**0.000**
Encoder-decoder	C2C	0.414	0.202	0.794	0.794	0.537	**0.001**	0.060	0.261
	R2C	0.563	0.324	0.765	**0.000**	0.657	**0.000**	0.237	0.376
	E2C	0.066	0.348	**0.000**	0.046	0.422	0.167	0.814	0.536
	MC2C	**0.004**	**0.002**	0.651	0.932	0.447	0.521	0.625	0.362
	MR2C	0.055	0.398	0.960	0.861	0.668	0.798	0.154	0.356
	ME2C	0.590	0.381	0.955	0.971	0.498	0.186	0.377	0.376

Values are compared between canonical SMILES representation attributions or random SMILES representation as compared to all other enumerated values. Bold values are p > 0.005

## Appendix E

**Entropy analyses**

See Figs. 9, 10

## Appendix F

**Carbon breakdown**

See Fig. 11

## Appendix G

**Relative importance**

See Figs. 12, 13, 14

## Code availability

Project home page: https://github.com/PeterHartog/augmented-xai. Operating system(s): Platform independent Programming language: Python 3 Other requirements: several open source python packages License: MIT. Any restrictions to use by non-academics: none.
